# Reducing False Arrhythmia Alarms Using Different Methods of Probability and Class Assignment in Random Forest Learning Methods

**DOI:** 10.3390/s19071588

**Published:** 2019-04-02

**Authors:** Krzysztof Gajowniczek, Iga Grzegorczyk, Tomasz Ząbkowski

**Affiliations:** 1 Department of Informatics, Faculty of Applied Informatics and Mathematics, Warsaw University of Life Sciences SGGW, 02-776 Warsaw, Poland; tomasz_zabkowski@sggw.pl; 2 Department of Physics of Complex Systems, Faculty of Physics, Warsaw University of Technology, 00-662 Warsaw, Poland; igagrze@gmail.com

**Keywords:** arrhythmia, false alarm, random forest, machine learning

## Abstract

The literature indicates that 90% of clinical alarms in intensive care units might be false. This high percentage negatively impacts both patients and clinical staff. In patients, false alarms significantly increase stress levels, which is especially dangerous for cardiac patients. In clinical staff, alarm overload might lead to desensitization and could result in true alarms being ignored. In this work, we applied the random forest method to reduce false arrhythmia alarms and specifically explored different methods of probability and class assignment, as these affect the classification accuracy of the ensemble classifiers. Due to the complex nature of the problem, i.e., five types of arrhythmia and several methods to determine probability and the alarm class, a synthetic measure based on the ranks was proposed. The novelty of this contribution is the design of a synthetic measure that helps to leverage classification results in an ensemble model that indicates a decision path leading to the best result in terms of the area under the curve (AUC) measure or the global accuracy (score). The results of the research are promising. The best performance in terms of the AUC was 100% accuracy for extreme tachycardia, whereas the poorest results were for ventricular tachycardia at 87%. Similarly, in terms of the accuracy, the best results were observed for extreme tachycardia (91%), whereas ventricular tachycardia alarms were the most difficult to detect, with an accuracy of only 51%.

## 1. Introduction

The population is aging worldwide, and the elderly need complex medical attention more often. With increasing numbers of elderly patients, machine and algorithm support is crucial for the smooth functioning of medical facilities. In intensive care units (ICU), patients’ lives are monitored by multiple bedside devices. Some of the signals recorded are electrocardiogram (ECG), respiratory effort, or pulsatile waveforms such as arterial blood pressure (ABP) and photoplehtysmogram (PPG). Based on these signals, heart arrhythmias are detected and alarms are generated. According to Aboukhalil et al. [1] and Drew et al. [2], the rates of false alarms in ICUs might be as high as almost 90%. Unnecessary alarms can negatively impact both ICU patients and medical stuff. Hence, reduction of false alarms would improve the quality of help provided for critical care patients. The task of reducing the false alarms starts at level of heart beat detection from the ECG signal, as no universal algorithms are resistant to all quality issues present in ECG recordings, e.g., noise or artefacts resulting from patient movements. The other problem occurs when the leads of electrodes fall off patients’ bodies. In this case, no heart beats are detected in ECG signals, just like in asystole; hence, an alarm would be generated. To avoid such errors, simultaneous analysis of additional signals is necessary. Generation of false alarms is a real and complex problem, which was examined in 2015 by organizers of the PhysioNet/Computing in Cardiology Challenge [3]. In this challenge, contestants were provided with 750 signals registered 5 min before the alarm generation and information about what type of arrhythmia caused the alarm. The alarms were triggered by five types of arrhythmia: asystole, bradycardia, tachycardia, ventricular tachycardia, and ventricular fibrillation or flutter. All the signals were analyzed by expert annotators and labelled as true or false.

Many of the well-performing algorithms in the challenge incorporated problem-specific rules. However, only a few of the researchers tried to apply general methods such as algorithms from the machine learning field. As such, in this study, we examined different methods of probability and class determining using random forest (RF). In particular, using the PhysioNet Challenge dataset that included the five types of the arrhythmia data, we aimed to answer the following research questions: (1) To what extent is it possible to reduce false arrhythmia alarms? (2) Which method best determines the probability/score from the classifier in terms of the performance on a new unseen dataset? (3) Which method best determines the class label from the classifier’s probability for classifying the new observation on the new unseen dataset?

The remainder of this paper is organized as follows: Section 2 provides an overview of the similar research problems for both reducing false arrhythmia alarms and RF. In Section 3, the theoretical framework of the RF algorithm is presented. In Section 4, model performance measures are presented. Section 5 describes theoretical aspects of the probability and class label assignment based on RF. Section 6 outlines the experiments and presents the discussion of the results. The paper ends with concluding remarks in Section 7.

## 2. Related Works

### 2.1. Arrhythmia Detection

In general, an arrhythmia occurs when the heartbeat is irregular, but some of them are defined only by the frequency of heart muscle contractions. Bradycardia occurs when there are less than 40 heart beats per minute (bpm). Tachycardia is diagnosed when a heart rate is over 140 bpm. No beat detected in at least four seconds is asystole. Such precise definitions make these arrhythmias easily detectable from an algorithmic point of view, but only when high quality data are available. In real life, the signals are often noisy and include artefacts, which is why it is so important to use robust algorithms for heart beat location within a signal. No golden standard algorithm yet exists for heart beat detection. The approaches used vary among scientific teams and companies. The most widely used method is the Pan-Tompkins algorithm [4], which is based on filtering techniques and precisely determines the location of the heartbeat but cannot handle different QRS complex morphologies (the part of ECG illustrating contraction of ventricles) [5], so the algorithm cannot analyze more complex arrhythmias such as ventricular tachycardia. To address this limitation, more advanced methods were developed [6,7]. When the quality of the ECG signal is poor but pulsatile waveforms or other signals are available, beats should be distinguished in such signals and used instead for arrhythmia identification. In the 2014 PhysioNet Challenge, methods were proposed to substitute the poor quality parts of ECG recordings with beats detected from other, usually pulsatile, signals such as arterial blood pressure (ABP) and plethysmogram (PLETH) [8,9,10]. In many cases, such operation allowed continuous monitoring of heart rate and its variability.

Ventricular tachycardia and ventricular flutter or fibrillation are arrhythmias that not only have abnormally fast rhythms but the morphology of the QRS complexes is different than in normal signals. Wider QRS complexes are characteristic of ventricular tachycardia apart from high heart rate (above 100 bpm), which distinguishes it from standard tachycardia. For ventricular flutter and fibrillation, heart rate is immeasurable, as properly developed QRS complexes do not occur in the signal. Here, the alarm would be generated when the oscillatory waveform characteristic for these arrhythmias is present for at least four seconds. Hence, in analysing such signals, the algorithm cannot rely solely on heart rate. The methods already proposed for detection of ventricular tachycardia include flutter/fibrillation approaches like autocorrelation analysis [11], wavelet transformations [12,13], sample entropy [14], machine learning methods with features derived from signal morphology and analysis of power spectrum [15], time-frequency representation images [16], empirical mode decomposition [17], or using the zero crossing rate combined with base noise suppression with discrete cosine transform and beat-to-beat intervals [18].

Analysing all mentioned arrhythmia types was the task for the 2015 PhysioNet/Computing in Cardiology Challenge by classifying all alarms generated in ICUs as true or false [3]. The approaches among participants varied significantly in terms of heartbeat detection methods, type of generated features, or length of signal considered. Many defined a set of heuristic rules as a final classifier. Contestants concentrated mostly on proper detection of heart beats and assessment of signal quality. Liu et al. [19] analysed 60 s of data prior to the alarm and, apart from generating standard features like QRS width or maximum heart rate, they compared the morphology of detected heart beats against the predefined template. Rodrigues and Couto [20] based their analysis on open-source beat detectors for most arrhythmias, but for ventricular fibrillation, they proposed their own solution, fitting a parabola on windows of 125 ms. They used information from both ECG and waveform signals, but ECG was given higher priority in this solution. Knowing that poor quality signals are often a main cause of alarm generation, Plesinger et al. [21], as a first step of the analysis, annotated parts of the signals as INVALIDS. Such segments were not totally excluded from further analysis and number of invalid samples were used as one of the features. The signals were then tested for regularity and if the result was negative, the test for specific arrhythmia was performed. This approach meets the logical assumption that normal signals should be the easiest to determine. Features were created as a result of Hilbert and Fourier transformations and descriptive statistics. A more unconventional approach was proposed by Hoog Antink et al. [10]. Instead of generating features easily understandable from physiological point of view, they analysed the self-similarity of the signal based on autocorrelation and beat-to-beat estimation. They adopted image processing methods such as two-dimensional (2D) Fourier transformation and principal component analysis to reduce dimensionality. The final decision was made with multiple machine learning methods. Among the solutions, two approaches used the random forest (RF) algorithm. Asadi et al. [22] firstly concentrated on assessment of signal quality. Then, based on the beats detected from both ECG and pulsatile signals, they generated a set of features that concentrated mostly on the regularity of the beats. Having this set of features describing the signal for each arrhythmia type, a separate RF classifier was applied for each. Srivastava et al. [23] presented a two-step approach. First, RF was implemented for parameters based on combinations of features obtained for ECG, PPG, and ABP. Then, some specific thresholds were set for parameters solely from pulsatile waveforms. The ensemble of these two steps produced a final algorithm.

### 2.2. Random Forest as a Machine Learning Algorithm

RF is an ensemble supervised machine learning technique that emerged in the beginning of the 21st century. Machine learning techniques have applications in many areas of statistical data analysis, which can be broadly classified as descriptive or predictive. Descriptive data analyses concentrate rather on describing the data (e.g., probability distributions) or grouping data into categories using the unsupervised paradigm. The most commonly solved problems in this context are: (1) clustering, which includes algorithms such as K-means, hierarchical clustering [24], and grade data analysis [25]; (2) dimensionality reduction, algorithms such as principal component analysis (PCA) and non-negative matrix factorization [26]; and (3) association and sequential rule mining, which includes algorithms such as Apriori, Eclat, CM-SPADE, and PrefixSpan [27].

Predictive data analysis involves exploring past data and generating conclusions or trends for future prediction. Predictive data analysis is related to the classical approach of model building using the supervised paradigm. The goal in supervised learning is to teach a function that maps an input to an output based on example input-output pairs. It infers a function from labeled training data consisting of a set of training examples. A supervised learning algorithm analyzes the training data and produces an inferred function that can be used for mapping new examples (from validation or test datasets). An optimal scenario allows the algorithm to correctly determine the response/outcome for unknown instances. This requires the learning algorithm to generalize from the training data to unseen situations in a reasonable way. Standard supervised learning problem can be generalized as: (1) standard supervised learning, which includes algorithms such as support vector machine (SVM), naïve Bayes, decision trees, k-nearest neighbor, boosting, random forest, or artificial neural network (ANN) [28]; (2) semi-supervised learning, including algorithms such as artificial neural networks or graph-based methods [29]; and (3) structured prediction algorithms, such as Bayesian networks or random field [30].

Standard supervised learning can be further divided into two categories in terms of the nature of the response variable [31]: (1) classification, which involves the problem of identifying to which of a set of predefined class/categories an observation belongs; and (2) regression, which is the problem of predicting a continuous quantity output.

In the context of the classification problem (due to the nature of the Challenge), an ensemble consists of a set of individually trained classifiers whose predictions are combined for classifying an instance. Many researches have shown that an ensemble is often more accurate than any single classifier in the ensemble [32]. In particular, we are of the opinion that the RF algorithm produces one of the best accuracies to date in many practical applications and has important advantages over the other techniques in terms of ability to handle highly non-linear biological data, robustness to noise, tuning simplicity (compared to other ensemble learning algorithms), and opportunity for executing parallel processing. Bagging [33] and boosting [34] are two popular methods for producing ensembles. These methods use re-sampling techniques to obtain different training sets for each of the classifiers. Bagging stands for “bootstrap aggregating”, which works on the concept of bootstrap samples, where each sample is generated from original dataset by sampling with replacement. The multiple classifiers generated in bagging are independent of each other [35]. In boosting, weights are assigned to each sample from the training dataset. They are generated sequential such that one classifier is generated in a single iteration. For generating classifier weights, training samples are updated based on classification results of classifier from previous iteration. The classifiers generated by boosting are dependent on each other [35].

The theoretical and the empirical research related to ensemble have shown that an ideal ensemble consists of highly correct classifiers that disagree as much as possible [35]. Generally, there are four approaches for building ensembles of diverse classifiers [36]: (1) combination level: design different combiners; (2) classifier level: use different base classifiers; (3) feature level: use different feature subsets; and (4) data level: use different data subsets.

To construct a good ensemble model, some improvements in RF have been introduced, which will be explained further. Boinee et al. [37] proposed meta learning techniques that are based on the concept that RF is the base classifier. Meta random forest incorporates both well-established concepts: bagging and boosting. Robnik-Šikonja et al. [38] proposed the ReliefF algorithm to evaluate attributes in the pre-processing step; quality estimates are used as weight for selecting subsamples of attributes at each level of the tree. This helps decrease the correlation between the attributes while maintaining the strength. Tsymbal et al. [39] suggested three different techniques for improving the voting scheme based on performance of local predictors: Dynamic Selection (DS), Dynamic Voting (DV), and Dynamic Voting with Selection (DVS). Bernard et al. [40] discussed a Forest algorithm in which the number of features considered in each split is randomly selected at each node during the tree induction process. To develop RF, usually many trees are required to increase the stability of the model and reduce the prediction errors. Unfortunately, a huge number of trees makes the forest uninterpretable. To resolve this problem, Wang et al. [41] proposed a shrinkage method to reduce the number of trees while simultaneously maintaining a similar level of accuracy. Xuanfu et al. [42] proposed an algorithm called BAGA that generates the ensemble using combination of bagging and genetic algorithm techniques, so that individual classifiers are determined at execution time. In dynamic random forests [40], individual base trees are added in a dependent manner rather than an independent approach. A new tree is added in the forest by evaluating of the sub-forest already built, thus taking an adaptive approach.

## 3. State-of-the-Art Random Forest

### 3.1. Decision Tree as a Base Algorithm

In a classification context, there is a training sample (training dataset D) of n observations on a class variable Y, which takes values 1, 2,…,k, (in this study k=2) and p predictor variables, $X_{1},\ldots ,\;X_{p}$. The goal is to find a model (DT) for predicting the values of Y from new X values [43]. In theory, the solution is simply a partition of X space into k disjoint sets, $A_{1},\ldots ,\;A_{k}$, such that the predicted ($\hat{Y}$) value of Y is j if X belongs to $A_{j}$ for j=1, 2,…,k. Classification tree methods yield rectangular sets $A_{j}$ by recursively partitioning the data set one X variable at a time.

One of the oldest and commonly used decision tree algorithm is the classification and regression tree (CART) [44], employing a measure of node impurity based on the distribution of the observed Y values in the node by splitting a node by exhaustively searching over all X and S for the split {X∈S} that minimizes the total impurity of its two child nodes, defined by the Gini Index [45]:

(1) $$I_{G}=1-\overset{k}{\underset{j}{\sum }}p_{j}^{2},$$

where $p_{j}$ denotes the estimated probability that an observation in $A_{j}$ belongs to class j. If X takes ordered values, the set S is an interval of the form (−∞,c]. Otherwise, S is a subset of the values taken by X. The process is applied recursively to the data in each child node. Splitting stops if the relative decrease in impurity is below a pre-specified threshold. Algorithm 1 provides the pseudocode for the basic steps.
**Algorithm 1**: Decision tree algorithm pseudocode.**input**: List of all explanatory variables (X), training dataset (D) **output**: Decision tree (DT) /1/ Start at the root node /2/ **for each**X in X
**do** /3/ find the set S that minimizes the sum of the node impurities in the two child nodes  in Equation (1) and choose the split $\left\{X^{*}\in S^{*}\right\}$ that produces the minimum overall X and S /4/ **end**
/5/ **if** stopping criterion is reached **then do** /6/ stop /7/ **else do** /8/ apply step 2 to each child node in turn /9/ **end** /10/ **return *DT***


There are various stopping criteria controlling the growth of the tree such as the minimum number of observations that must exist in a node in order for a split to be attempted, the minimum number of observations in any terminal node (leaf), or the maximum depth of any node of the final tree. To achieve good generalization ability (i.e., small error rate on the unseen examples), the tree first grows in an overly large size and then it is pruned to a smaller size to minimize the misclassification error. CART employs 10-fold (default) cross-validation.

Each branch of the tree ends in a terminal node, each terminal node is uniquely defined by a set of rules, and each observation falls into exactly one terminal node. Finally, each of the leaves assigns the probability of an observation belonging to a particular class. Many computer software implementations simply return the class label by taking class with the highest probability. For example, in a binary classification problem, if the probability ≥ 0.5, then Class1; otherwise, Class2.

### 3.2. Standard Random Forest Algorithm

The original [46] RF algorithm operates by constructing many decision trees during training and outputting the prediction of the individual trees, i.e., class label. RF overcomes decision tree’s habit of overfitting their training dataset.

The training algorithm for RF applies the general technique of bootstrap aggregating [33], also called bagging, to the base learners (decision tree). Given a training dataset D of size n, bagging generates m new training sets $D_{i}$, each of size $n^{\prime }$, by sampling from D uniformly and with replacement. By sampling with replacement, some observations may be repeated in each $D_{i}$. If $n^{\prime }=n$, then for large n, the dataset $D_{i}$ is expected to have the fraction 1−1e≈63.2% of the unique examples of D, the rest being duplicates. The m models are fitted using the above m bootstrap samples and combined by majority voting:

(2) $${\hat{Y}}_{RF}=\mathrm{majority}\;\mathrm{voting}\;{\left\{{\hat{Y}}_{i}\right\}}_{1}^{m},$$

where $\hat{Y}$ is the predicted class from the ith tree in the forest. For the draw, a class for a particular observation is assigned randomly. This bootstrapping procedure leads to better model performance because it decreases the variance in the model without increasing the bias. This means that although the predictions of a single tree are highly sensitive to noise in its training dataset, the average of many trees is not as long as the trees are not correlated.

The above procedure describes the original bagging algorithm for decision trees. The RF algorithm has an additional modification—it uses a modified Algorithm 1, called a random decision tree that selects a random subset of the features mtry at each candidate split in the learning process. This process is sometimes called feature bagging. The reason for doing this is the correlation of the trees in an ordinary Bootstrap sample: if one or a few features are very strong predictors of the response variable (target output), these features will be selected in many of the m trees, causing them to become correlated. Typically, for a classification problem with p features, $floor\left(\sqrt{p}\right)$ features are used in each split. All the steps for building the RF are summarized in Algorithm 2.
**Algorithm 2**: Random Forest algorithm pseudocode.**input**: Number of Trees (m), random subset of the features (mtry), training dataset (D) **output**: random forest (RF) /1/ RF is empty /2/ **for each**
i to m
**do** /3/ $D_{i}$ = Bootstrap Sample (D) /4/ $DT_{i}$ = Random Decision Tree ($D_{i}$, mtry) /5/ RF = $RF\cup DT_{i}$ /6/ **end** /7/ **return**
RF

Each tree within the forest is built to its maximum size, i.e., without pruning. The evaluation of the model performance on the training dataset is often replaced by an Out of Bag (OOB) sample. This is a method of measuring the prediction error on the remaining 36.8% observations not observed in the bootstrap sample (In Bag). OOB is the mean prediction error using only the trees that did not have a particular observation in their bootstrap sample.

## 4. Model Performance Measures

Evaluating models based on machine learning algorithms is an essential part of any project. We used different types of evaluation metrics, which are briefly described below.

### 4.1. Confusion Matrix and Score Function

Confusion matrix, as the name suggests, produces a matrix as the output and describes the complete performance of the model. Consider a binary classification problem [47]. Samples can belong to one of the two classes: yes or no. A classifier predicts a class for a given input sample. In this context, there are four important terms, as shown in Table 1:

- True Positives (TP): the cases that predicted yes and the actual output was also yes,
- True Negatives (TN): the cases that predicted no and the actual output was no,
- False Positives (FP): the cases that predicted yes and the actual output was no, and
- False Negatives (FN): the cases that predicted no and the actual output was yes.

For the purpose of the Challenge and based on the above table, the score measure was computed. The score measure was designed to treat FN, genuinely life-threatening events that the program considered unimportant, especially harshly, and is defined as:

(3) $$\mathrm{Score}=\frac{100\times \left(TP+TN\right)}{TP+FP+TN+5\times FN}.$$

Each classifier returns a probability of belonging to a particular observation in the positive class (in this case, yes). This probability is then discretized into two possible outcomes based on the same threshold/cutoff value, which is usually set to 0.5. It is a natural approach to assign the most probable class, i.e., if probability ≥ 0.5, then yes; otherwise, no. Unfortunately, in many situations, this threshold is not optimal (optimality should be defined in advance), but some methods can determine the best cutoff point. These are outlined in Section 5.

### 4.2. Receiver Operating Curve (ROC) and Area Under the ROC

Area Under the Curve (AUC) is one of the most widely used metrics for evaluation. AUC is used for binary classification problem (extensions for multiclass classification problems also exists). The AUC of a classifier is equal to the probability that the classifier will rank a randomly chosen positive example higher than a randomly chosen negative example [48]. Before defining AUC, two basic terms should be explained: true positive rate (TPR or sensitivity) is defined as TP/(FN+TP). Sensitivity corresponds to the proportion of positive data points that are correctly considered positive with respect to all positive data points. False positive rate (FPR or specificity) is defined as FP/(FP+TN). Specificity corresponds to the proportion of negative data points that are mistakenly considered positive with respect to all negative data points. For the purpose of the Challenge, complementary measures, such as true negative rate, defined as TNR=1−FPR, are easily determined.

**Figure 1:**
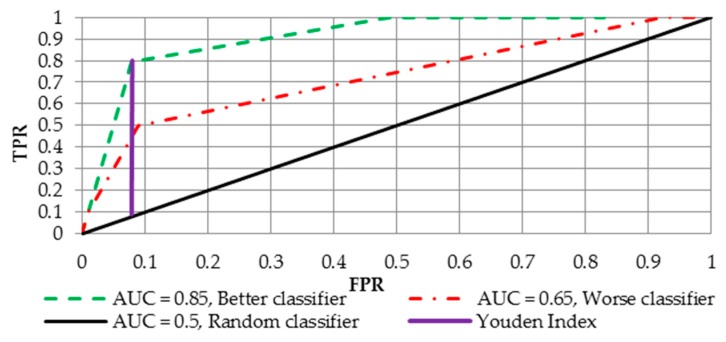
The receiver operating curve (ROC) and its possible variants.

FPR and TPR both have values in the range [0, 1]. FPR and TPR are both computed at threshold values, such as (0.00, 0.02, 0.04, …., 1.00) and a graph was drawn as shown in Figure 1. AUC is the area under the curve of the plot FPR vs. TPR at different points in [0, 1].

As evident, AUC has a range of [0, 1]. The greater the value, the better the performance of the model is. 

### 4.3. Optimal Treshold for Class Determination

Since the ROC is considered to be the best global measure assessing the effectiveness of a model, it is a good starting point for searching for an optimal cutoff point. To benefit from the optimal score threshold, Youden’s J statistic [49] was employed. The optimal cutoff is defined as (see the purple line in Figure 1):

(4) max(TPR+(1−FPR)).

The Youden Index is the point on the ROC that is farthest from line of equality (diagonal line in Figure 1). The main aim of the Youden Index is to maximize the difference between TPR and FPR. The value of J for a continuous test can be located by searching plausible values where the sum of sensitivity and specificity is maximized. The Youden Index is the most commonly used criterion (compared to other measures used in this context) because this index reflects the intension to maximize the correct classification rate and is easy to calculate [50].

## 5. Methods of Probability and Class Determining

**Figure 2:**
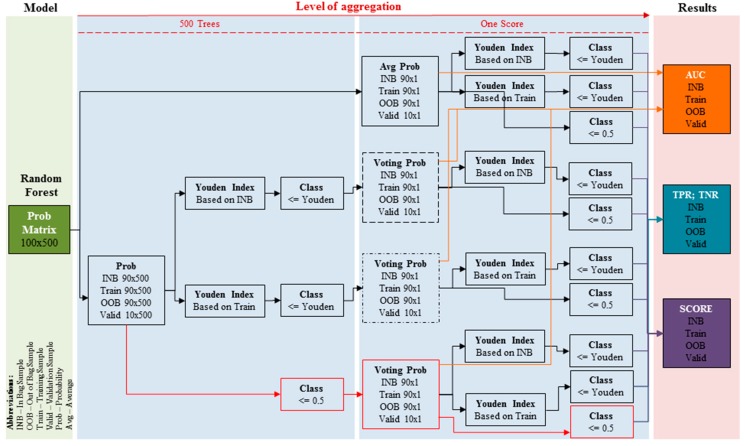
Possible approaches for probability and class determination.

After the RF is built, every computer implementation (packages like *randomForest* or *ranger* in *R* environment) returns both a matrix with an estimated probability for each observation and a tree within the forest, and a matrix that indices whether a particular observation was in the In Bag (1 flag) or the OOB (0 flag) sample for a particular tree. After combining the information in these matrixes, the estimated probability for a particular observation can be determined in four different ways (Figure 2): (1) averaging (Avg Prob table); (2) majority class voting, where the cutoff for class determining is derived based on the In Bag sample (dashed Voting Prob table); (3) majority class voting, where the cutoff for class determining is derived based on the training sample (dotted-dashed Voting Prob table); and (4) majority class voting, where the cutoff is set to 0.5 (red Voting Prob table). For the sake of this example, assume that an entire dataset that includes 100 observations is divided into a training sample (90 observations) and a validation sample (10 observations). Also assume that the RF includes 500 trees, which means that the analyzed matrix is 100 × 500. 

Now consider the first method to determine probability (for the training examples) based on averaging. Table 2 presents the probability of an observation belonging to the positive class with the indicator of whether an observation is in the In Bag (INB, 1 flag on the bright red color) or the Out of Bag (OOB, 0 flag on the bright green color) sample. The final probability is determined as an average probability for all the trees (directly without any inner steps like in other approaches) where an observation was in INB (bright red) or in OOB (bright green). In the third case, the average probability is simply taken based on all the trees (white). The probability for the validation sample is also taken from all trees e.g., based on the first row ((0.5 + 0.7 + 0.1 + 0.1)/4) is 0.35.

Now consider the second method to determine the probability of the training examples based on majority voting (Table 3). Consider that the class label for each tree is determined based on the cutoff for probability set to 0.5, i.e., if probability ≤ 0.5 then “NO” else “YES”. This is equivalent to the standard Breiman’s implementation of the RF (red line in Figure 2). As in the previous example, voting is completed in three different ways. Take the second observation: there are two trees where it is in the INB sample. Unfortunately, these trees predict two different classes this is why the final probability is set at 0.5. In the OOB sample, Tree3 and Tree4 predict class “no”; therefore, the final probability of being positive is 0.

This simple example shows that the estimated probabilities could be very different. Considering other approaches to setting the optimal cutoff (based on the Youden Index) for determining the class label for majority voting produces many different and diverse solutions. The variety of approaches for determining of the class probability implicates 10 different methods for deriving the final class for a particular observation. These approaches incorporate determining the final class either based on the cutoff set to 0.5 or derived from the Youden Index. 

In summary, the probability (first level of aggregation) and the final class (second level of aggregation) assignment to each observation can be derived from four main approaches and 10 sub-approaches (Figure 2):

(1) Probability: averaging all the probabilities from all the trees within the forest (Avg Prob; upper solid line table);
  (a) Class: cutoff (Youden Index) for class determination is derived based on the In Bag sample (abbreviation: Prob INB),
  (b) Class: cutoff (Youden Index) for class determination is derived based on the training sample (abbreviation: Prob Train),
  (c) Class: cutoff for class determination is set at 0.5 (abbreviation: Prob 0.5).
(2) Probability: majority class voting where the cutoff for class determining is derived based on the In Bag sample (upper-middle dashed line table; abbreviation: Voting Prob INB);
  (a) Class: cutoff (Youden Index) for class determining is derived based on In-Bag sample (abbreviation: Vote INB INB),
  (b) Class: cutoff for class determination is set to 0.5 (abbreviation: Vote INB 0.5).
(3) Probability: majority class voting where the cutoff for class determining is based on the training sample (lower-middle dotted-dashed line table; abbreviation: Voting Prob Training);
  (a) Class: cutoff (Youden Index) for class determining is based on the training sample (abbreviation: Vote Train Train),
  (b) Class: cutoff for class determination is set to 0.5 (abbreviation: Vote Train 0.5).
(4) Probability: majority class voting where the cutoff is set to 0.5 (bottom red line table, equivalent to the standard RF; abbreviation: Voting Prob 0.5);
  (a) Class: cutoff (Youden Index) for class determination is derived based on the In Bag sample (abbreviation: Vote 0.5 INB),
  (b) Class: cutoff (Youden Index) for class determination is derived based on the training sample (abbreviation: Vote 0.5 Train),
  (c) Class: cutoff for class determination is set to 0.5 (abbreviation: Vote 0.5 0.5).

After producing both the probabilities and the class labels, various model performance measures for each dataset and the approach can be computed.

## 6. Empirical Analysis

### 6.1. Feature Vector

As mentioned in the introduction, the data included two ECG channels, at least one pulsatile waveform, either arterial blood pressure (ABP) or plethysmogram, and respiratory effort. The contestants were provided with 750 recordings as a training set. The distribution of the signals among arrhythmias and whether the alarms were true or false are presented in Table 4. The numbers presented in the table vary slightly from those in the paper describing the challenge data in detail [3]. After the 2015 Challenge ended, there was a discussion that some of the arrhythmia type labels were assigned incorrectly. This resulted in organizers consulting the experts and reloading the data with corrected arrhythmia type labels. Those data were analyzed in this paper: each recording was 5 min long and recorded with a sampling frequency of 250 Hz. Then, each recording was pre-filtered against noise with mains notch filters and a FIR band pass filter (0.05–40 Hz) [3].

The first step of the data processing was beat detection in both ECG and pulsatile signals, which enabled later proper signal selection. Beat detection in ECG was performed as proposed by Eerikäinen et al. [51], based on the low-complexity R-peak detector [52]. The beat locations were determined with the use of an adaptive threshold on a convolution of ECG and single wavelet. The algorithm used for beat detection in ABP and PLETH was wabp [53], an open source algorithm from PhysioNet [3]. Then, the quality of the signals was assessed using the F1 score [51]. In this method, both ECG leads and available pulsatile waveforms were compared to each other. In window of 14–16 s before the alarm (depending on the arrhythmia type), signals were compared beat by beat to verify whether their locations matched. If so, beats were marked as true positive (TP); otherwise, as false positive (FP) or false negative (FN) depending on which of the compared signals an additional beat was detected. Then the F1-score was calculated as F1 = 2 TP/(2 TP + FP + FN). A result of 1 signified that detected beats matched among all signals; a result of 0 meant that found annotations were totally different. Based on the F1 scores, two signals were chosen for the next step, which was feature computation.

The set of features for the arrhythmias was generated in line with the schema presented by Eerikäinen [51]. For each arrhythmia, features were generated based on the clinical definition provided by challenge organizers [3]. For asystole, extreme bradycardia, and extreme tachycardia, only the features described by heart rate and inter-beat intervals were considered 14–16 s before the alarm [51]. For ventricular tachycardia and ventricular flutter or fibrillation, such metrics were insufficient due to their characteristics (Section 2.1.). Hence, the modified spectral purity index (SPI) [51] approach was implemented and the minimum and maximum of the calculated SPI were incorporated as features.

### 6.2. Numerical Implementation

All numerical experiments were conducted in the *R* environment [54] using a personal computer equipped with an Intel Core i5-2430M 2.4 GHz processor (2 CPU × 2 cores), 8 GB RAM, and the Ubuntu 16.04 LTS operating system. The core of the entire analysis is the *ranger* package [55], implementing the state-of-the-art Breiman’s RF. This software is written in *C++* and *R* and is a fast implementation of RF, especially suited for high dimensional data. To conduct the research, many wrapper functions working on the output from the *ranger* package were written. For instance, these functions extract the class probability matrix or In Bag/Out of Bag matrix for each observation and tree. The optimal threshold for class determining based on the Youden Index was calculated using the *pROC* library [56].

The estimates for the performance measures for the training and validation samples were produced with k-fold cross-validation. The number of k sets was set to 10 when there were more than 10 samples in the smaller class. Otherwise, k was set to the size of the smaller class to ensure that there was at least one sample from both of the classes (9 for extreme tachycardia and 6 for ventricular fibrillation or flutter). The k sets were generated so that the class distribution in every set represented the class distribution of the entire dataset using stratified sampling based on the *createFolds* function implemented in the *caret* library [57]. All further results are presented as an average over *k*-folds with the standard errors of the estimates.

### 6.3. Detailed Results for AUC and Score for Various Samples

**Figure 3:**
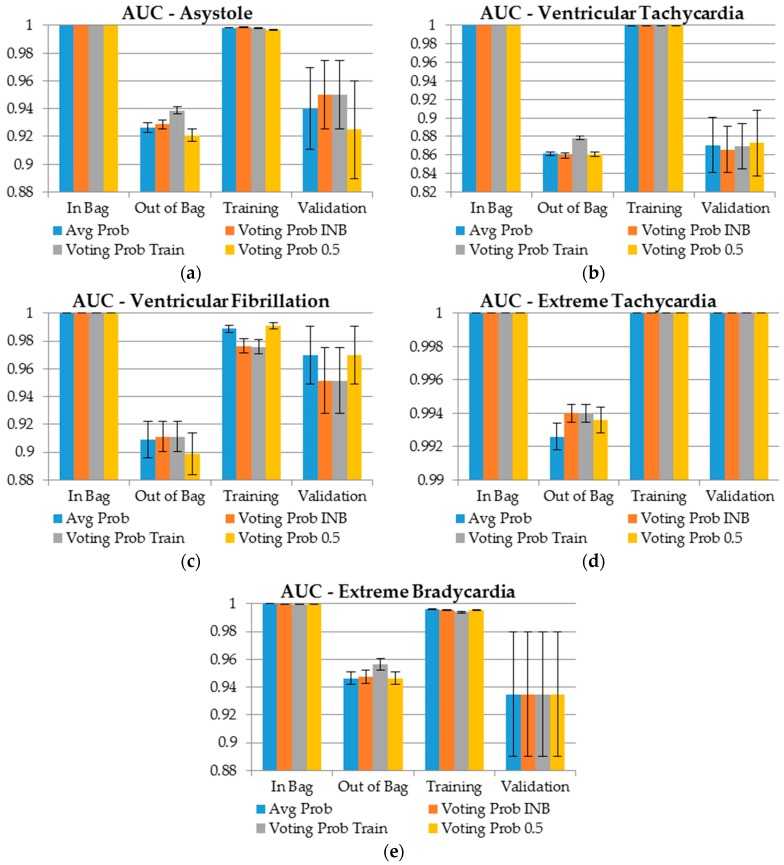
AUC results for each type of arrhythmia and data sample in terms of method of probability assignment. (**a**) Asystole, (**b**) Ventricular Tachycardia, (**c**) Ventricular Fibrillation, (**d**) Extreme Tachycardia, (**e**) Extreme Bradycardia.

**Figure 4:**
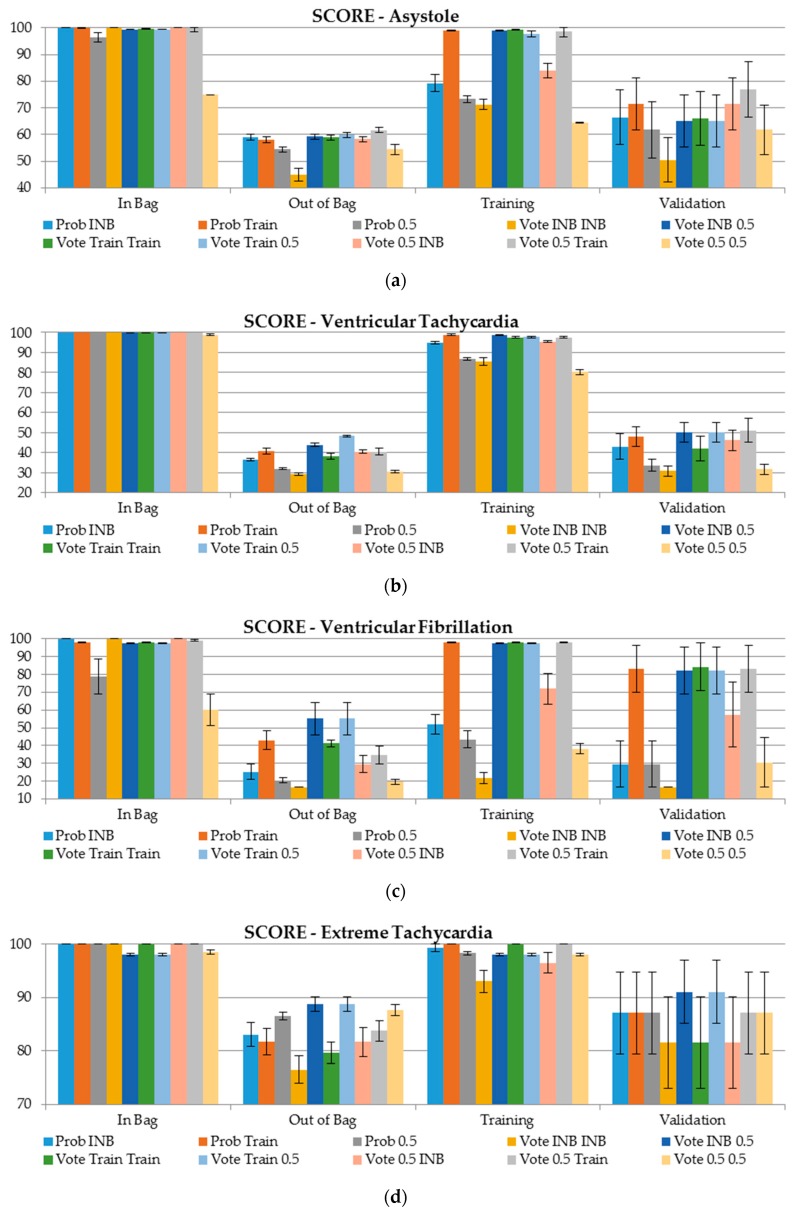

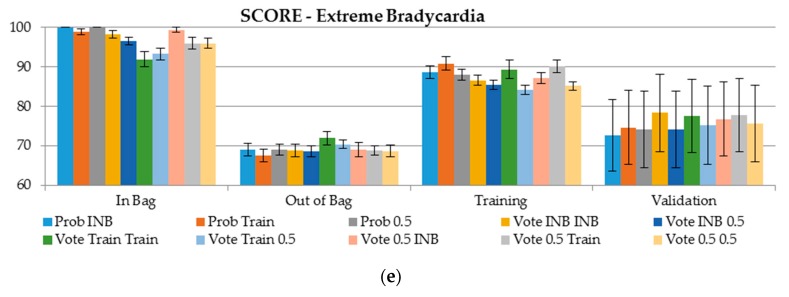
Score results for each type of arrhythmia and sample in terms of method of class label determining. (**a**) Asystole, (**b**) Ventricular Tachycardia, (**c**) Ventricular Fibrillation, (**d**) Extreme Tachycardia, (**e**) Extreme Bradycardia.

The classification performance of each approach for probability and class determination was evaluated with AUC, TPR, TNR, and a challenge score (Equation (3)) within the training, In Bag, Out of Bag, and validation datasets. The results are presented in Figure 3 and Figure 4 and in Appendix A. Each approach has its own color in the figure with whiskers representing standard error of estimation. Standard errors for TPR and TNR are presented in brackets. 

Because each tree within the forest is built to its maximum depth, each tree affects very few observations on a particular leaf, which results in inpurity being almost 0, i.e., perfect classification. This directly translates into almost perfect results in terms of AUC for the In Bag sample for each type of arrhythmia (Figure 3). Since the training dataset is approximately 63.2% similar to the In Bag dataset, the results for this sample were also reletively accurage. The acuracy was especially high for ventricular tachycardia and extreme tachycardia. Due to dependency on the learning process, the aforementioned samples should be considered only as additional information. Since in machine learning the goal is generalization of the knowledge, the OOB and validation samples should be used for assement of the models. 

We observed that, in most of the cases, the highest AUC for the OOB sample was achieved using the entire training sample for the Youden Index determination and then simple majority voting (gray color). This occurred because the cutoff was determined also with respect to the knowledge partialy (36.8%) contained in this sample. This approach has relatively smaller standard errors of the estimates. In terms of the validation sample, we observed that the results for each method for extreme tachycardia and extreme bradycardia were equal. The results for extreme tachycardia were almost perfect: there were only nine negative cases (Table 4), and due to the stratified sampling, each fold contained only one case of this type. Generally, ventricular tachycardia is the type of arrhythmia that is relatively the most difficult to detect since the AUC for both unseen samples during the training process is the smallest, i.e., ranging between 0.86 and 0.87, when for other types of arrythmia, the AUC is far greater than 0.9.

In terms of the score measured in Equation (3), any case incorrectly classified as negative (FN) is punished five times more greatly than other mistakes (FP). So, even one mistake has a considerable influence on this measure. This was observed even for the In Bag sample, especially where, at some stage of the final class determination (either for the probability or for the finall class label), the constant cutoff set to 0.5 was employed, for instance for asystole or ventricular fibrillation. 

For the previous measure, the relatively lowest results were produced for ventricular tachycardia (score ranging between 30 and 50 for the validation sample). Extreme tachycardia was the type of arrhythmia with the highest score values for the validation sample (80–90) and with the smallest differences between each method of class label determination. Since the positive class in ventricular fibrillation or flutter is relatively rare and the average value was estimated based on six folds containing only one case, the results for the unseen sample are very different for each approach of class determination.

To compare our results with the those achived by other researchers [11,21,22,23,51], Appendix A (Table A1, Table A2, Table A3, Table A4 and Table A5) provides the detailed results of TPR and TNR for each type of arrhythmia and the data sample in terms of methods for class assignment. We compared the top five results from the 2015 PhysioNet/Computing in Cardiology Challenge (after the follow-up phase) and two other algorithms using RF for final classification with our best solution, as shown in Table A6. The results shown in the table were calculated for the training set only, as we did not have access to the hidden test set. The reason for providing the results from the follow-up phase of the Challenge, instead of the official phase, is that they were calculated on the data with labels revised after the official phase, which we used as well. The proposed algorithm produces the best results for both TPR and TNR for extreme tachycardia and ventricular flutter fibrillation, and is among the top results for asystole, extreme tachycardia, and extreme bradycardia. Most of the algorithms had the lowest false alarm suppression for ventricular flutter/fibrillation and ventricular tachycardia, which, apart from different morphology of the signals, might be a result of an unbalanced training set for these types of arrhythmia. Of the algorithms using the RF method, the Asadi approach had noticeably lower results and although Srivastava’s solution produced an impressive TPR for all arrhythmias, apart from ventricular flutter/fibrillation, TN ratios were rather low. Our comparison among different approaches proved that the proposed RF classifier often produces better results than already existing methods and might contribute to solving the problem of the high rate of false arrhythmia alarms in ICUs.

### 6.4. Aggregated Ranks for AUC and Score Based on the Validation Sample

The results presented in Section 6.3. provide a broad overview of the performance of each approach. However, analyzing the results on bar charts is a challenging task and our goal was to present the aggregated results with a synthetic measure. Therefore, to answer the question of which approach is the most appropriate for probability and for class assignment, a “global” synthetic measure had to be developed. For this purpose, we used ranks that would require a two-step procedure. In the first step, “local” ranks for each type of arrhythmia were derived, and secondly, based on these local ranks, one global rank was created.

**Figure A1:**
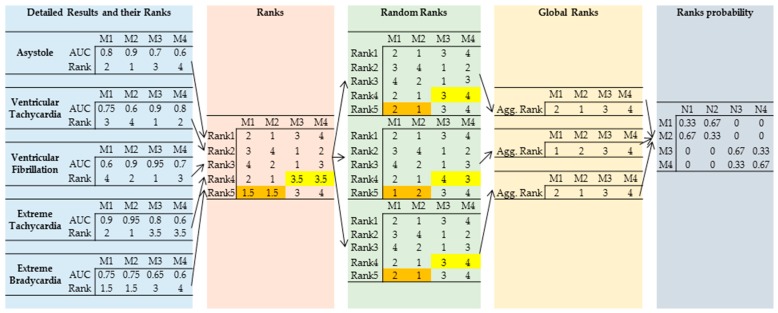
The approach to derive global ranks (N1–N4) for each method (M1–M4) for probability assignment and each arrhythmia type based on the AUC measure on the validation sample. Orange and yellow highlight the equal ranks; these were subject to 3-fold random rank assignment.

Ranks in the first step were created using the *rank()* function in *R*. This function returns integers for each value where, in this case, the first position is assigned the highest value in terms of AUC or score results. In some cases, the results might be equal (a so-called tie), which considerably impacts the second step of this procedure. To overcome this issue, positions with equal values at the corresponding indices were randomly assigned. In the second step, local ranks are aggregated to the global rank using the *RankAggreg* library [58]. Rank aggregation is an essential approach for aggregating multiple preferences. Rank aggregation could be cast in the framework of an optimization problem, which needs some objective function. In this context, the aim was to find a super list (rank) which would be as close as possible to all individual ordered lists (ranks) simultaneously. This is a simple and intuitive requirement and takes the form:

(5) $$\theta \left(\sigma \right)=\overset{m}{\underset{i=1}{\sum }}w_{i}d\left(\sigma ,L_{i}\right),$$

where $L_{i}$ is the ith ordered list (4 for AUC ranks and 10 for score ranks); σ is a proposed ordered list of length $k=\left|L_{i}\right|$, i.e., 4 for AUC ranks and 10 for score ranks; $w_{i}$ is the importance weight associated with list $L_{i}$ (in this case all weights are equal); and d is a distance measure based on the Spearman correlation (for better understanding please see Figure A1 in the Appendix B).

The main goal of ranks aggregation is to find $\sigma^{*}$ that minimize the total distance between $\sigma^{*}$ and ith *L*:

(6) $$\sigma^{*}=\arg\min\overset{m}{\underset{i=1}{\sum }}w_{i}d\left(\sigma ,L_{i}\right).$$

Due to the relatively small number of the investigated lists, rank aggregation was performed using the exhaustive search, i.e., generating all possible ordered lists and finding the list with the minimum value of the above objective function [58]. For details of the aggregation process, refer to Appendix B. This approach works for relatively small problems only and should not be attempted if k is relatively large (k > 10); otherwise, methods like cross-entropy Monte Carlo and genetic algorithm are applied but these methods do not guarantee an optimal solution and both these algorithms are sensitive to the tuning parameters. 

As the first step incorporates some randomness in determining the final ranks, the results presented below (based on the validation sample) are created based on the simulation analysis. Local ranks in the first step were determined in a repetitive manner (1000 repetitions; therefore, Table 5 and Table 6 show the distribution of the appearance of each approach at a particular position in the global rank (gray color palette indicates intensity from the highest in dark gray to the lowest in white). 

Table 5 shows that the highest AUC values for each type of arrhythmia are associated with the approach where the probability for each observation is determined using majority class voting, where the class is derived based on the cutoff set to 0.5 (Voting Prob 0.5). This resulted in 53.83% of the cases the majority class voting delivering the highest AUC value (first ranking position: N1). The second place (rank: N2, 51.25%) was the approach where the final probability is determined using averaging (Avg Prob) of all the probabilities from all the trees. The third and the fourth place were assigned to majority class voting where the cutoff for class determination is derived based on training or In Bag samples, respectively.

Regardless of the size of the analyzed problem, Table 6 reveals relatively stable results. The first and the last place are known for sure in 100% of the cases. The best score was received by the approach where, firstly, the probability for each observation is determined using majority class voting where the cutoff for the class is set to 0.5 and, secondly, the final class is determined using the cutoff derived based on the training sample. Last place is the approach where, at both stages, the cutoff is determined based on the In Bag sample.

The most unstable results were related to probability averaging in the first stage and the Youden Index determination based on the In Bag sample during the second stage (with four possible positions); majority class voting where the cutoff for the class is set to 0.5 during the first stage and, during the second stage, the cutoff is also set to 0.5 (with three possible positions with relatively similar chances). 

## 7. Conclusions

The detection of false arrhythmia alarms in Intensive Care Units is a challenging task for classification algorithms as a number of triggers for false alarms are possible, including the noises arising from motion artifacts, patient sweating and movement, and temporary machine malfunctions, such as detachment of electrodes and sensors.

The novelty of our research was demonstrated through the design of a synthetic measure that helps to manage complex classification problems whenever the classification tasks pertain to a set of classification tasks (e.g., predicting different arrhythmia types) and/or several methods to determine the probability and the class labels. Our proposed measure helps to leverage classification results in ensemble methods as it indicates the decision path leading to the best results in terms of the AUC measure or the global accuracy (score).

In particular, we focused on the application of RF to the classification of five arrhythmia alarms as true or false. Additionally, we examined four different methods for probability aggregation and 10 methods for class assignment in RF, as these impact the accuracy of the classification.

This synthesis of the approaches is applicable for probability and class assignment and addresses the literature gap and draws attention to one of the important aspects of ensemble classifiers. The classification performance of the RF algorithm was evaluated with the AUC, True Positive Rate, True Negative Rate, and a challenge score [3] using the Out of Bag and validation datasets. The following results were obtained:

(1) Ventricular tachycardia is the arrhythmia for which the false alarms are the most difficult to detect since AUC values for both unseen samples (OOB and validation) have the lowest ranges, between 0.86 and 0.87;
(2) Extreme tachycardia arrhythmia false alarms are by far the easiest to detect as the AUC values are close to 1 for both OOB and validation datasets;
(3) The AUC value is the greatest for the OOB sample when the probability is selected using majority class voting where the cutoff for class assignment is derived based on the training sample;
(4) In terms of the score measure, the validation dataset delivers better results for false alarms detection in comparison with Out of Bag; however, the results are biased with higher standard errors;
(5) In terms of the score measure, ventricular tachycardia false alarms are difficult to capture as the scores are lower than 50, and slighly better scores are produced using the validaton dataset;
(6) For ventricular fibrillation, the scores obtained on the validation dataset are 30 points better than using OOB (score 50 vs. 80, respectively). This was observed when the cutoff for the class assignment was derived based on the training sample, or the cutoff for class determining was set to 0.5 (Figure 4).

For the aggregated results with ranks, the following results were observed:

(1) In 53.83% of the cases, the majority class voting with the cutoff set to 0.5 (Voting Prob 0.5) produced the highest AUC value (first ranking position: N1);
(2) The best score (first ranking position: N1) was observed when the probability for each observation was determined using majority class voting with the cutoff for the class set to 0.5. The final class was determined using the cutoff derived based on the training sample (Vote 0.5 Train). This approach produces the highest score on the validation dataset.

The results for the aggregated level indicate that application of the proper approach to determine probability and the class label is an important task that may affect the classification accuracy. We think that the problem of probability and class assignment in RF is valid, and therefore, our study can be extended further with the application of other machine learning methods in ensemble mode or multiple different learning algorithms to produce better predictive performance. This may lead to further study on algorithms’ diversity and the effects of varying ensemble size on classification accuracy.

## Figures and Tables

**Table 1:** Confusion matrix for binary classification.

		Predicted Value	
		Positive (P)	Negative (N)
**Real value**	**Positive (P)**	True Positive (TP)	False Negative (FN)
	**Negative (N)**	False Positive (FP)	True Negative (TN)

**Table 2:** Example of Probability matrix and In Bag matrix with estimated probability for each observation based on the average probability.

No.	Tree1	Tree2	Tree3	Tree4	Avg Prob INB	Avg Prob OOB	Avg Prob Train
**1**	0.6(1)	0.7(0)	0.8(1)	0.2(0)	0.70	0.45	0.58
**2**	0.5(1)	0.7(1)	0.1(0)	0.1(0)	0.50	0.10	0.35
**3**	0.1(0)	0.9(1)	0.4(1)	0.8(0)	0.65	0.45	0.55

**Table 3:** Example of Probability matrix and In Bag matrix with estimated probability for each observation based on the majority voting.

No.	Tree1	Tree2	Tree3	Tree4	Vote Prob INB	Vote Prob OOB	Vote Prob Train
**1**	YES (1)	YES (0)	YES (1)	NO (0)	1.00	0.50	0.75
**2**	NO (1)	YES (1)	NO (0)	NO (0)	0.50	0.00	0.25
**3**	NO (0)	YES (1)	NO (1)	YES (0)	0.50	0.50	0.50

**Table 4:** Arrhythmia types and alarm distribution used in the 2015 Challenge.

Arrhythmia Type	NO	YES
Ventricular Tachycardia	252	89
Asystole	100	22
Extreme Tachycardia	9	131
Ventricular Fibrillation or Flutter	52	6
Extreme Bradycardia	43	46

**Table 5:** Distribution of global ranks for probability assignment in terms of the AUC measure observed for the validation sample.

Approach	Ranks			
	N1	N2	N3	N4
Avg Prob	27.86%	51.25%	15.38%	5.51%
Voting Prob INB	9.03%	13.14%	22.77%	55.06%
Voting Prob Train	9.28%	13.80%	51.89%	25.03%
Voting Prob 0.5	53.83%	21.81%	9.96%	14.40%

**Table 6:** Distribution of global ranks for probability assignment in terms of the score observed on the validation sample.

Approach					Rank					
	N1	N2	N3	N4	N5	N6	N7	N8	N9	N10
Prob INB	0.00%	0.00%	0.00%	0.00%	0.00%	53.06%	23.73%	5.25%	17.95%	0.00%
Prob Train	0.00%	25.04%	74.26%	0.70%	0.00%	0.00%	0.00%	0.00%	0.00%	0.00%
Prob 0.5	0.00%	0.00%	0.00%	0.00%	0.00%	0.00%	0.00%	61.91%	38.09%	0.00%
Vote INB INB	0.00%	0.00%	0.00%	0.00%	0.00%	0.00%	0.00%	0.00%	0.00%	100.00%
Vote INB 0.5	0.00%	74.96%	23.82%	0.00%	0.61%	0.61%	0.00%	0.00%	0.00%	0.00%
Vote Train Train	0.00%	0.00%	0.35%	0.00%	7.97%	38.62%	53.06%	0.00%	0.00%	0.00%
Vote Train 0.5	0.00%	0.00%	1.58%	59.19%	37.74%	1.49%	0.00%	0.00%	0.00%	0.00%
Vote 0.5 INB	0.00%	0.00%	0.00%	40.11%	53.68%	6.22%	0.00%	0.00%	0.00%	0.00%
Vote 0.5 Train	100.00%	0.00%	0.00%	0.00%	0.00%	0.00%	0.00%	0.00%	0.00%	0.00%
Vote 0.5 0.5	0.00%	0.00%	0.00%	0.00%	0.00%	0.00%	23.20%	32.84%	43.96%	0.00%

## Appendix A

**Table A1:** True Positive Rate (TPR) and True Negative Rate (TNR) for Asystole.

	TPR In Bag	TNR In Bag	TPR Out of Bag	TNR Out of Bag	TPR Training	TNR Training	TPR Validation	TNR Validation
**Prob INB**	**100(±0)**	**100(±0)**	77.2(±0.9)	93.9(±0.3)	89.9(±1.7)	99(±0.1)	75(±8.3)	94(±2.2)
**Prob Train**	**100(±0)**	99.9(±0.1)	77.2(±0.9)	91.6(±0.4)	**100(±0)**	98.2(±0.3)	80(±8.2)	92(±2)
**Prob 0.5**	98.5(±0.8)	**100(±0)**	72.2(±1)	96.9(±0.2)	86.3(±0.8)	**100(±0)**	70(±8.2)	**97(±1.5)**
**Vote INB INB**	**100(±0)**	**100(±0)**	60.5(±3)	**97.7(±0.3)**	84.8(±1.3)	**100(±0)**	61.7(±6.6)	**97(±1.5)**
**Vote INB 0.5**	**100(±0)**	99(±0.2)	78.3(±0.8)	91.3(±0.4)	**100(±0)**	97.8(±0.3)	75(±8.3)	91(±2.3)
**Vote Train Train**	**100(±0)**	99.2(±0.2)	77.3(±0.9)	93.8(±0.7)	**100(±0)**	98.4(±0.2)	75(±8.3)	93(±2.1)
**Vote Train 0.5**	**100(±0)**	99(±0.2)	78.3(±0.7)	93.1(±0.5)	99.5(±0.5)	97.9(±0.3)	75(±8.3)	91(±2.3)
**Vote 0.5 INB**	**100(±0)**	**100(±0)**	77.2(±0.9)	91.9(±0.3)	92.9(±1.3)	97.6(±0.3)	80(±8.2)	92(±2)
**Vote 0.5 Train**	**100(±0)**	99.9(±0.1)	**80.8(±1.5)**	89(±0.5)	**100(±0)**	97.1(±0.3)	**85(±7.6)**	90(±2.6)
**Vote 0.5 0.5**	87.3(±0.9)	**100(±0)**	72.2(±1)	97.1(±0.2)	79.8(±1.5)	**100(±0)**	70(±8.2)	**97(±1.5)**

**Table A2:** TPR and TNR for Ventricular Tachycardia.

	TPR In Bag	TNR In Bag	TPR Out of Bag	TNR Out of Bag	TPR Training	TNR Training	TPR Validation	TNR Validation
**Prob INB**	**100(±0)**	**100(±0)**	51.8(±1.1)	91.1(±0.5)	98(±0.3)	99.6(±0.1)	56.7(±6.3)	90.1(±1.5)
**Prob Train**	100 (±0)	**100(±0)**	58.9(±2.3)	88.4(±0.8)	99.6(±0.2)	99.4(±0.1)	65.6(±4.5)	88.1(±1.9)
**Prob 0.5**	**100(±0)**	**100(±0)**	42.5(±0.9)	94.4(±0.2)	94(±0.4)	**100(±0)**	42.9(±5.7)	94.5(±0.9)
**Vote INB INB**	**100(±0)**	**100(±0)**	36.8(±1.4)	**95.4(±0.2)**	93.3(±1.1)	**100(±0)**	38.1(±5.2)	**95.3(±1.2)**
**Vote INB 0.5**	**100(±0)**	99.8(±0.1)	64.2(±0.9)	86.1(±0.4)	**100(±0)**	97.3(±0.2)	**68.9(±4.6)**	85.3(±1.6)
**Vote Train Train**	**100(±0)**	**100(±0)**	53.4(±2.3)	92.1(±0.5)	99.1(±0.2)	99.3(±0.1)	55.6(±6.4)	89.7(±1.5)
**Vote Train 0.5**	**100(±0)**	99.6(±0.1)	**69.3(±0.6)**	86.1(±0.4)	99.8(±0.2)	96.7(±0.2)	**68.9(±4.6)**	85.3(±1.6)
**Vote 0.5 INB**	**100(±0)**	**100(±0)**	58.7(±1.3)	88.5(±0.5)	98.3(±0.2)	99.3(±0.1)	63.3(±4.7)	87.7(±1.8)
**Vote 0.5 Train**	**100(±0)**	**100(±0)**	58.4(±2.4)	88(±0.8)	99.1(±0.2)	99.4(±0.2)	67.8(±5.4)	88.9(±1.4)
**Vote 0.5 0.5**	99.5(±0.2)	**100(±0)**	39.5(±1.1)	95.1(±0.2)	90.5(±0.8)	**100(±0)**	39.3(±5.3)	**95.3(±1)**

**Table A3:** True Positive Rate and True Negative Rate for Ventricular Fibrillation or Flutter.

	TPR In Bag	TNR In Bag	TPR Out of Bag	TNR Out of Bag	TPR Training	TNR Training	TPR Validation	TNR Validation
**Prob INB**	**100(±0)**	**100(±0)**	23.3(±9.5)	96.2(±0.5)	66.7(±6.7)	98.9(±0.8)	16.7(±16.7)	98.2(±1.9)
**Prob Train**	**100(±0)**	96.6(±0.5)	56.7(±8)	95.4(±0.6)	**100(±0)**	96.2(±0.5)	**83.3(±16.7)**	94.2(±2.6)
**Prob 0.5**	86.7(±6.7)	**100(±0)**	13.3(±4.2)	97.7(±0.8)	56.7(±6.1)	99.6(±0.4)	16.7(±16.7)	98.2(±1.9)
**Vote INB INB**	**100(±0)**	**100(±0)**	0(±0)	**99.6(±0.4)**	13.3(±8.4)	**100(±0)**	0(±0)	100(±0)
**Vote INB 0.5**	**100(±0)**	95.4(±0.6)	**70(±6.8)**	94.2(±1)	**100(±0)**	95.4(±0.6)	**83.3(±16.7)**	92.4(±3.8)
**Vote Train Train**	**100(±0)**	96.2(±0.5)	56.7(±3.3)	96.2(±0.5)	**100(±0)**	96.2(±0.5)	**83.3(±16.7)**	96.3(±2.3)
**Vote Train 0.5**	**100(±0)**	95.4(±0.6)	**70(±6.8)**	94.2(±1)	**100(±0)**	95.4(±0.6)	**83.3(±16.7)**	92.4(±3.8)
**Vote 0.5 INB**	**100(±0)**	**100(±0)**	33.3(±9.9)	95.4(±0.6)	83.3(±6.1)	98.1(±0.9)	50(±22.4)	98.2(±1.9)
**Vote 0.5 Train**	**100(±0)**	98.1(±0.9)	43.3(±10.9)	93.1(±1.2)	**100(±0)**	96.2(±0.5)	**83.3(±16.7)**	94.2(±2.6)
**Vote 0.5 0.5**	73.3(±6.7)	**100(±0)**	10(±4.5)	98.9(±0.8)	50(±4.5)	**100(±0)**	16.7(±16.7)	**100(±0)**

**Table A4:** True Positive Rate and True Negative Rate for Extreme Tachycardia.

	TPR In Bag	TNR In Bag	TPR Out of Bag	TNR Out of Bag	TPR Training	TNR Training	TPR Validation	TNR Validation
**Prob INB**	**100(±0)**	**100(±0)**	99.2(±0.1)	69.4(±4.5)	**100(±0)**	98.6(±1.5)	**99.2(±0.8)**	77.8(±15.6)
**Prob Train**	**100(±0)**	**100(±0)**	99.2(±0.1)	66.7(±4.9)	**100(±0)**	**100(±0)**	**99.2(±0.8)**	77.8(±15.6)
**Prob 0.5**	**100(±0)**	**100(±0)**	99.2(±0.1)	76.4(±1.5)	99.3(±0.1)	**100(±0)**	**99.2(±0.8)**	77.8(±15.6)
**Vote INB INB**	**100(±0)**	**100(±0)**	**99.7(±0.2)**	54.2(±4.9)	**100(±0)**	86.1(±4.1)	**99.2(±0.8)**	66.7(±17.7)
**Vote INB 0.5**	99.2(±0.1)	**100(±0)**	98.8(±0.2)	**83.3(±3.1)**	99.2(±0.1)	**100(±0)**	98.5(±1.1)	**88.9(±11.8)**
**Vote Train Train**	**100(±0)**	**100(±0)**	99.2(±0.1)	62.5(±3.8)	**100(±0)**	**100(±0)**	**99.2(±0.8)**	66.7(±17.7)
**Vote Train 0.5**	99.2(±0.1)	**100(±0)**	98.8(±0.2)	**83.3(±3.1)**	99.2(±0.1)	**100(±0)**	98.5(±1.1)	**88.9(±11.8)**
**Vote 0.5 INB**	**100(±0)**	**100(±0)**	99.2(±0.1)	66.7(±5.4)	**100(±0)**	93.1(±3.9)	**99.2(±0.8)**	66.7(±17.7)
**Vote 0.5 Train**	**100(±0)**	**100(±0)**	99.2(±0.1)	70.8(±3.8)	**100(±0)**	**100(±0)**	**99.2(±0.8)**	77.8(±15.6)
**Vote 0.5 0.5**	99.4(±0.2)	**100(±0)**	99.1(±0)	79.2(±2.2)	99.2(±0.1)	**100(±0)**	**99.2(±0.8)**	77.8(±15.6)

**Table A5:** True Positive Rate and True Negative Rate for Extreme Bradycardia.

	TPR In Bag	TNR In Bag	TPR Out of Bag	TNR Out of Bag	TPR Training	TNR Training	TPR Validation	TNR Validation
**Prob INB**	**100(±0)**	**100(±0)**	86.7(±0.8)	87.9(±1.3)	96.1(±0.7)	94.6(±0.6)	82.5(±8.7)	87(±4.7)
**Prob Train**	99.5(±0.3)	**100(±0)**	85.5(±1.1)	88.1(±1.6)	**96.4(±1)**	**97.9(±0.8)**	82.5(±8.7)	91(±3.7)
**Prob 0.5**	**100(±0)**	**100(±0)**	86.5(±0.7)	88.9(±1.2)	95.4(±0.8)	96.6(±0.7)	82.5(±8.7)	88.5(±3.9)
**Vote INB INB**	99.3(±0.4)	**100(±0)**	86.7(±0.9)	87.1(±1.4)	94.9(±0.6)	95.6(±0.7)	**85(±8.9)**	88.5(±3.9)
**Vote INB 0.5**	98.6(±0.4)	**100(±0)**	86(±0.7)	89.4(±1.1)	94(±0.6)	97.4(±0.4)	82.5(±8.7)	88.5(±3.9)
**Vote Train Train**	96.9(±1)	97.9(±0.8)	**89.1(±1.5)**	85.2(±2.4)	95.9(±1.3)	96.6(±1.2)	**85(±8.9)**	88.5(±3.9)
**Vote Train 0.5**	97.1(±0.7)	**100(±0)**	86.7(±0.5)	**91.2(±0.9)**	93.5(±0.6)	96.6(±0.6)	82.5(±8.7)	90.5(±3.9)
**Vote 0.5 INB**	99.8(±0.2)	**100(±0)**	87(±1)	86.6(±1.3)	95.4(±0.7)	94.8(±0.9)	**85(±8.9)**	87(±4.7)
**Vote 0.5 Train**	98.3(±0.7)	99.7(±0.3)	86.7(±0.7)	87.1(±1.8)	96.1(±0.8)	97.7(±0.6)	84.5(±8.9)	91(±3.7)
**Vote 0.5 0.5**	98.3(±0.5)	**100(±0)**	85.7(±0.9)	90.4(±0.9)	93.7(±0.5)	**97.9(±0.3)**	82.5(±8.7)	**93(±3.6)**

**Table A6:** Comparison of our best solution (Vote 0.5 training), top 5 solutions from the 2015 PhysioNet/Computing in Cardiology Challenge (after the follow-up phase), and two other solutions that used RF in their approach. All the results were calculated for the training set.

Method	Asystole		Ventricular Tachycardia		Ventricular Flutter or Fibrillation		Extreme Tachycardia		Extreme Bradycardia	
	TPR	TNR	TPR	TNR	TPR	TNR	TPR	TNR	TPR	TNR
Gajowniczek et al. (Vote 0.5 training)	**100**	97	99	**99**	**100**	**96**	**100**	**100**	96	98
Plesinger et al. [3]	**100**	68	80	80	**100**	85	99	78	91	60
Krasteva et al. [3]	**100**	**99**	81	77	**100**	**96**	**100**	**100**	98	91
Kalidas et al. [3]	**100**	86	84	85	**100**	65	**100**	89	**100**	93
Hoog Antink et al. [3]	**100**	89	93	85	**100**	92	**100**	**100**	**100**	77
Eerikäinen et al. [3]	90	90	89	63	67	94	98	88	93	83
Asadi et al. [22]	39	49	68	85	78	89	87	82	56	60
Srivastava et al. [23]	**100**	89	**100**	67	83	88	**100**	67	**100**	72
